# The gut-immune axis in primary immune thrombocytopenia (ITP): a paradigm shifts in treatment approaches

**DOI:** 10.3389/fimmu.2025.1595977

**Published:** 2025-06-12

**Authors:** Xuejun Guo, Ke Wang, Qianhui Liu, Natalia Baran, Wenxue Ma

**Affiliations:** ^1^ Department of Hematology, Puyang Oilfield General Hospital Affiliated with Xinxiang Medical College, Puyang, Henan, China; ^2^ Department of Hematology and Central Hematology Laboratory, Inselspital, Bern University Hospital, University of Bern, Bern, Switzerland; ^3^ Institute of Hematology and Transfusion, Department of Experimental Hematology, Warsaw, Poland; ^4^ Department of Medicine, Sanford Stem Cell Institute, Moores Cancer Center, University of California San Diego, San Diego, CA, United States

**Keywords:** primary immune thrombocytopenia (ITP), gut-immune axis, immune dysregulation, microbiota-based therapy, platelet homeostasis

## Abstract

Primary immune thrombocytopenia (ITP) is an autoimmune disorder characterized by platelet destruction and impaired production, leading to bleeding risk. While immunosuppressive therapies are standard, many patients experience relapses or refractory disease, highlighting the need for novel approaches. Emerging evidence suggests the gut microbiota plays a role in immune regulation, yet its impact on ITP remains unclear. Dysbiosis has been linked to immune dysfunction in other autoimmune diseases, but whether it drives or results from immune dysregulation in ITP is debated. This review explores the gut-immune axis in ITP, focusing on microbiota-driven immune modulation, cytokine signaling, and platelet homeostasis. We assess microbiota-targeted interventions, including fecal microbiota transplantation (FMT), probiotics, and dietary modifications, while addressing key controversies and knowledge gaps. Advances in microbiome sequencing and artificial intelligence may facilitate personalized interventions. Standardizing microbiota-based diagnostics and validating their efficacy in clinical trials are crucial for their integration into ITP management. Bridging these gaps may lead to microbiota-driven strategies that enhance immune regulation and improve patient outcomes.

## Highlights

- The gut-immune axis influences ITP pathogenesis and platelet homeostasis.
- Dysbiosis disrupts immune regulation and drives disease progression.
- FMT, probiotics, and dietary interventions offer potential ITP therapies.
- Microbiome sequencing and AI may advance personalized treatments.
- Standardization and clinical validation are crucial for microbiota-based strategies.

## 1 Introduction

Primary immune thrombocytopenia (ITP) is an acquired autoimmune disorder characterized by a persistent low platelet count due to immune-mediated platelet destruction and impaired platelet production (1, 2). While immunosuppressive therapies, such as corticosteroids and intravenous immunoglobulin (IVIG), are commonly used, their effectiveness varies, and long-term management remains challenging (3, 4). In particular, the frequent need for alternative therapeutic strategies in refractory cases underscores the need for a deeper understanding of ITP pathogenesis and the development of novel treatment approaches.

Traditionally, ITP is associated with autoantibody-mediated platelet destruction, where antibodies target platelet surface glycoproteins such as GPIIb/IIIa and GPIb/IX (5–7). However, emerging evidence reveals a more complex pathophysiology involving dysregulated T-cell responses, pro-inflammatory cytokines, and defective megakaryopoiesis. An imbalance between regulatory T cells (Tregs) and effector T cells (Th1 and Th17) drives persistent inflammation and immune-mediated platelet destruction, underscoring the multifaceted nature of ITP pathogenesis (8, 9). Notably, a reduction or dysfunction of Tregs is associated with the induction of ITP, whereas their expansion or restoration is considered immunoprotective, restoring immune tolerance and suppressing autoreactive responses against platelets (10).

Beyond these immune mechanisms, recent research suggests that gut microbiota plays a crucial role in shaping immune responses in ITP (11). The gut-immune axis, which governs interactions between intestinal microbiota and systemic immunity, has been implicated in various autoimmune diseases, including systemic lupus erythematosus and rheumatoid arthritis (RA) (12–14). Dysbiosis, an imbalance in gut microbial composition, has been shown to drive immune dysregulation, promote inflammation, and influence hematologic conditions (15–17). However, the precise role of the gut microbiota in ITP pathogenesis remains unclear, presenting a significant knowledge gap.

A key controversy center on whether alterations in the gut microbiota are a consequence of immune dysfunction in ITP or an independent driver of disease progression (18). Additionally, while microbiota-targeted interventions such as fecal microbiota transplantation (FMT) and probiotics have shown promise in other autoimmune conditions, their therapeutic potential in ITP remains largely unexplored (19, 20). These gaps highlight the need for further investigation into the mechanisms linking gut dysbiosis to ITP pathogenesis and treatment response.

This review therefore comprehensively analyzes the gut-immune axis in ITP, critically evaluating current knowledge and identifying key unanswered questions. We explore the potential of microbiota-targeted therapies to restore immune homeostasis, highlight areas of consensus and controversy, and propose directions for future research. By synthesizing available evidence and pinpointing knowledge gaps, we aim to advance the understanding of gut microbiota’s role in ITP and its implications for novel therapeutic strategies.

## 2 The gut-immune axis: a new perspective in ITP pathogenesis

Recent advancements in immunology and microbiome research have revealed the intricate interplay between the gut microbiota and systemic immune regulation (21, 22). This relationship is particularly relevant in autoimmune disorders such as primary ITP, where immune dysregulation leads to platelet destruction. The gut-immune axis serves as a dynamic interface between microbial communities and immune homeostasis, influencing inflammation and hematologic balance (22). Although disruptions in this axis have been implicated in several autoimmune diseases, their precise role in hematologic diseases like ITP remains poorly understood, underscoring significant gaps in current knowledge (19, 23).

### 2.1 Overview of the gut microbiota and immune system interactions

The gut microbiota, comprising a diverse and dynamic ecosystem of bacteria, viruses, fungi, and archaea, interacts closely with the immune system to maintain immune homeostasis. Gut-associated lymphoid tissue (GALT) plays a pivotal role in sensing microbial antigens and orchestrating immune responses (24). Dendritic cells (DCs) sample microbial metabolites and antigens, directing the differentiation of naïve T cells into Tregs or effector T cells (Th1 and Th17) (25, 26). This interaction promotes immune tolerance while maintaining a controlled inflammatory response.

However, the precise role of the gut microbiota in modulating hematologic immune responses remains controversial (22, 27). While some studies suggest that gut microbial communities influence immune regulation through cytokine production, others propose that the immune system primarily shapes microbiota composition (28, 29). This bidirectional relationship underscores the complexity of gut-immune interactions and necessitates further investigation in the context of ITP.

**Figure 1:**
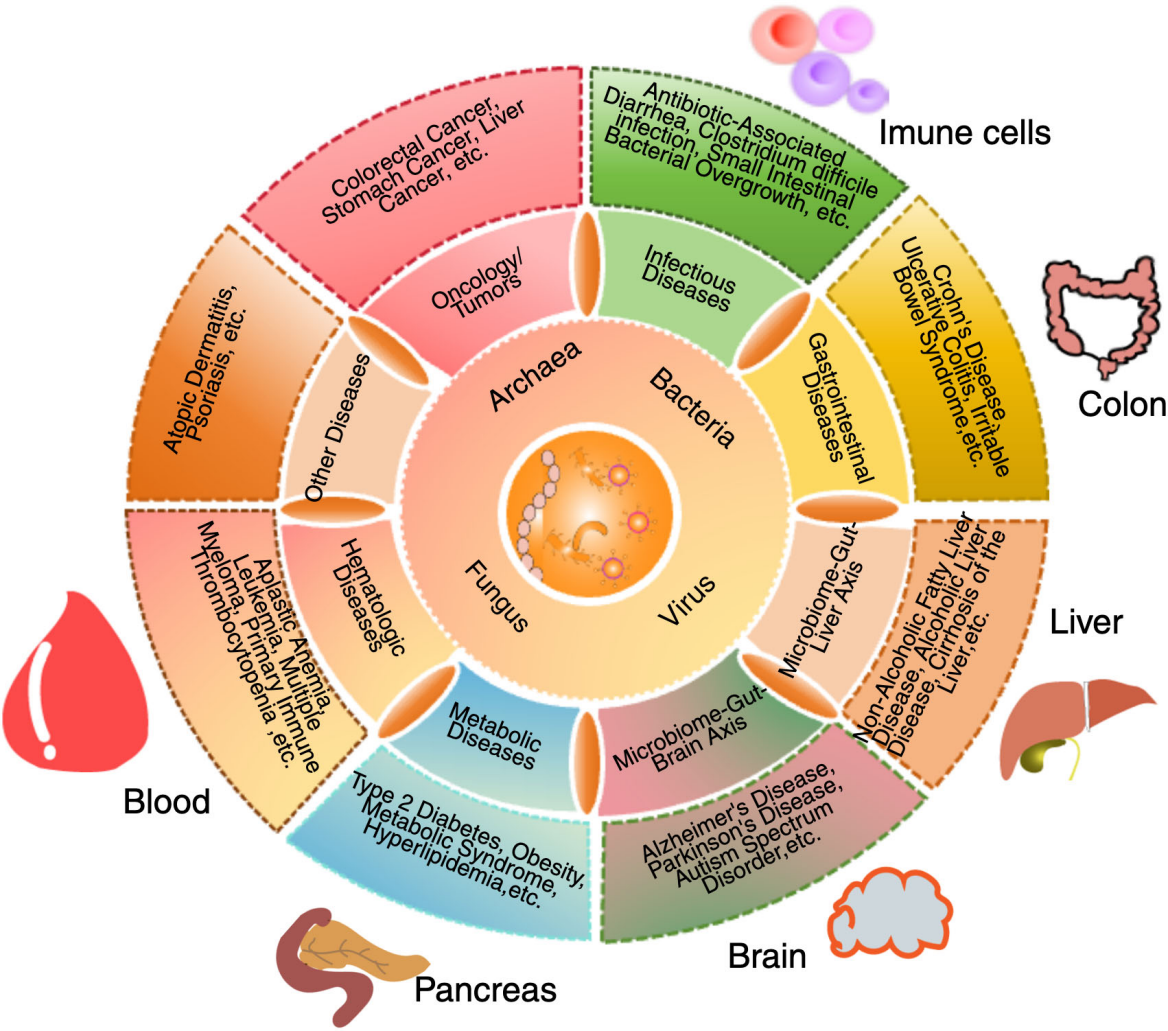
Interactions between gut microbiota, systemic organs and the immune system. The gut microbiota plays a central role in regulating immune function, metabolism, and overall systemic health through interactions with multiple organ systems. This schematic illustration depicts how bacteria, viruses, fungi, and archaea influence host physiology by modulating immune responses, metabolic processes, and disease development. Dysbiosis has been implicated in various conditions, including autoimmune diseases, neurodegenerative disorders, metabolic syndromes, and inflammatory diseases. Key organ systems affected by alterations in microbiota include the immune system, colon, liver, and pancreas. In immune system, the gut microbiota regulates Tregs, Th1/Th17 responses, and inflammation. In the colon, microbial communities influence gut barrier integrity and are associated with inflammatory bowel diseases. In the liver, the gut-derived metabolites affect metabolic conditions such as non-alcoholic fatty liver disease (NAFLD). In the pancreas, microbiota alterations have been associated with type 2 diabetes and other metabolic disorders. In the bloodstream, microbial dysbiosis is linked to hematologic conditions, including immune thrombocytopenia (ITP). In the brain, microbiota-derived metabolites have been implicated in neurological conditions such as Alzheimer’s disease, depression, and neuroinflammation. By modulating microbial composition and function, the gut microbiota exerts local and systemic effects that contribute to immune homeostasis, disease pathogenesis, and potential therapeutic interventions.

The intricate interactions between the gut microbiota and the immune system, including the influence of both commensal and pathogenic microbes in shaping immune tolerance, modulating inflammatory responses, and regulating systemic immune regulation, is critical to understanding hematologic disorders such as ITP. These mechanisms are visually summarized in **Figure 1**.

### 2.2 Mechanisms of gut microbiota in immune homeostasis and the role of B cells in ITP

The gut microbiota plays a pivotal role in immune regulation through multiple interconnected mechanisms, influencing both systemic and hematologic immune responses (12). Key pathways include microbial metabolite production, pattern recognition receptor (PRR) signaling, and maintenance of intestinal barrier integrity, each impacting immune homeostasis and ITP disease progression (30).

One essential pathway involves the production of microbial metabolites, particularly short-chain fatty acids (SCFAs) such as butyrate, propionate, and acetate, generated by commensal bacteria fermenting dietary fiber (31). SCFAs enhance regulatory T cell (Treg) differentiation, suppress inflammatory cytokines (IL-6, TNF-α, IFN-γ), and promote anti-inflammatory responses both systemically and within the gut (32). A reduction in SCFA-producing bacteria has been linked to impaired immune tolerance, heightened inflammation, and increased risk of autoimmunity, suggesting a potential link between dysbiosis and ITP pathogenesis (33, 34). Importantly, diminished Treg frequency or function is associated with ITP induction, whereas restoration or expansion of Tregs has been shown to confer protective effects by re-establishing immune homeostasis and attenuating platelet destruction (35).

Another critical mechanism is PRR signaling, where Toll-like receptors (TLRs) and nucleotide-binding oligomerization domain (NOD)-like receptors (NLRs) recognize microbe-associated molecular patterns (MAMPs) to distinguish commensal bacteria from pathogens (36, 37). This interaction normally regulates immune tolerance by limiting unnecessary immune activation (38). However, in gut dysbiosis, aberrant PRR signaling may trigger chronic immune activation, disrupting the balance between Tregs/effector T cells (Th1/Th17) and promoting a pro-inflammatory milieu that exacerbates platelet destruction in ITP (39, 40).

Furthermore, the gut microbiota maintains intestinal barrier integrity, preventing the translocation of microbial components into systemic circulation (41). Tight junction proteins (occludin, claudin, zonulin) regulate intestinal permeability, preventing leakage of bacterial endotoxins e.g., lipopolysaccharides (LPS) into the bloodstream (41, 42). Dysbiosis-induced compromise of the gut barrier can elevate circulating LPS levels, trigger systemic inflammation, and aberrantly activate monocytes and DCs factors that may contribute to immune dysregulation and platelet destruction in ITP (11, 43).

Although these mechanisms highlight the immunomodulatory potential of the gut microbiota, key controversies remain regarding its specific role in ITP (12, 44). For instance, SCFA’s effects on immune tolerance appear to be context-dependent, varying with disease state, microbial composition, and host genetic factors (45, 46). Similarly, while PRR signaling is crucial for immune surveillance, its dysbiosis-related overactivation has been associated with both immune suppression and chronic inflammation, resulting in conflicting findings in hematologic disorders (22). This lack of consensus underscores the need for further research to clarify how gut microbiota alterations influence platelet homeostasis and immune regulation in ITP (19, 47).

In addition to T cell-mediated pathways, B cells, particularly regulatory B cells (Bregs) have emerged as important players in maintaining immune tolerance in autoimmune disorders, including ITP (48). Bregs exert their immunosuppressive effects primarily through IL-10 production, which inhibits pro-inflammatory T cell responses and promotes Treg development (49). Dysregulation of Bregs has been observed in ITP patients, suggesting that impaired Breg function may contribute to loss of peripheral tolerance and heightened platelet destruction (50). Gut microbiota has been shown to modulate Breg development and function, likely via microbial metabolites and PRR signaling. Thus, altered microbial composition in ITP may impair Breg-mediated suppression of autoimmunity, further implicating the gut-immune axis in disease pathogenesis (51).

### 2.3 Dysbiosis and its impact on autoimmunity and hematologic diseases

Dysbiosis, defined as a disruption in gut microbial composition, has been implicated in numerous autoimmune and hematologic disorders (16, 52). Studies have demonstrated that conditions such as systemic lupus erythematosus (SLE), rheumatoid arthritis (RA), and multiple sclerosis (MS) exhibit characteristic microbial imbalances, often marked by an overrepresentation of pro-inflammatory species (e.g., Prevotella, Ruminococcus) and a concurrent depletion of beneficial bacteria (e.g., Bifidobacterium, Lactobacillus) (53, 54). These alterations are associated with immune dysregulation, excessive cytokine production, and chronic inflammation, all of which contribute to disease progression (55).

In hematologic disorders, gut dysbiosis has been increasingly associated with the breakdown of immune tolerance, platelet dysregulation, and systemic inflammation (19, 52). Certain bacterial taxa, such as *Enterobacteriaceae* and *Prevotella*, have been associated with an elevated pro-inflammatory Th17 response, promoting systemic inflammation and disrupting hematologic homeostasis (41, 56). Conversely, commensal bacteria like *Bifidobacterium* and *Lactobacillus* have been shown to enhance regulatory T cell (Treg) activity, mitigate excessive immune activation, and support immune balance (57, 58). The depletion of these beneficial microbes in patients with ITP suggests a potential role of the gut microbiota in modulating platelet homeostasis; however, direct causal relationships have yet to be confirmed (19, 47).

Beyond immune cell modulation, dysbiosis is associated with metabolic shifts that further influence immune responses (59, 60). A decrease in SCFA-producing bacteria, such as *Bacteroides* and *Firmicutes*, correlates with reduced Treg activity, impaired immune regulation, and increased inflammation, all of which are observed in autoimmune conditions (32, 61). Additionally, altered bile acid metabolism and tryptophan catabolism can modulate T cell differentiation, cytokine production, and systemic immune responses, potentially exacerbating platelet destruction in ITP (32, 62). However, whether these microbial and metabolic changes are a cause or consequence of immune dysregulation in ITP remains unclear, necessitating longitudinal studies and mechanistic research to establish causality.

A growing body of evidence suggests that distinct microbiota alterations are shared among various autoimmune diseases, highlighting common patterns of dysbiosis that may drive immune dysregulation across multiple conditions (12, 44). **Table 1** summarizes key microbial alterations in autoimmune diseases, including ITP, and their associated immune effects.

**Table 1:** Key microbiota alterations in autoimmune diseases and ITP.

Disease	Microbial changes	Immune impact	References
Systemic lupus erythematosus (SLE)	Decreased *firmicutes*, increased *bacteroidetes*	Elevated Th17 responses, decreased Tregs	(60, 63)
Rheumatoid arthritis (RA)	Increased *Prevotella copri*, reduced SCFA-producing bacteria	Pro-inflammatory cytokine production (IL-6, TNF-α)	(64, 65)
Multiple sclerosis (MS)	Reduced *akkermansia*, increased pro-inflammatory taxa	Enhanced Th1/Th17 responses	(66, 67)
Primary immune thrombocytopenia (ITP)	Decreased *bacteroides* and *firmicutes*, increased *enterobacteriaceae*	Impaired immune tolerance, platelet destruction	(19, 68)

### 2.4 Evidence linking gut microbiota alterations to ITP development and severity

Emerging research suggests that alterations in gut microbiota may contribute to the pathogenesis of ITP; however, findings remain inconsistent (18, 69). Comparative studies of the gut microbiomes of ITP patients and healthy controls have identified notable microbial differences (52, 70). Specifically, a reduction in SCFA-producing bacteria, such as *Bacteroides* and *Firmicutes*, may impair immune tolerance mechanisms, potentially exacerbating autoimmune responses (12, 46). Concurrently, an increase in pro-inflammatory taxa, including *Escherichia coli* and *Enterobacteriaceae*, has been associated with heightened Th17 responses and systemic inflammation (71). Additionally, experimental models suggest that antibiotic-induced dysbiosis can alter platelet counts and immune responses, reinforcing a potential link between gut microbiota and ITP progression (19, 72).

Despite these compelling findings, several critical gaps remain in the literature (11, 47, 72–74). Many studies are limited by small sample sizes, cross-sectional designs, and a lack of longitudinal analyses, making it difficult to establish causality between gut dysbiosis and ITP development (74). To clarify the causal relationship between gut dysbiosis and ITP, recent proposals emphasize the need for prospective longitudinal studies that track microbiota composition before and after disease onset or treatment (72). Additionally, mechanistic investigations using gnotobiotic mouse models may help elucidate how specific microbial taxa and metabolites influence immune regulation and platelet homeostasis (75, 76). Furthermore, while microbiota-targeted therapies such as FMT and probiotics have shown promise in other autoimmune diseases, their efficacy and applicability in ITP remain largely unexplored (77). Emerging studies indicate that microbiota-driven systemic metabolic changes can influence immune regulation in diseases beyond the gut, as seen in brain metastasis, where alterations in the gut microbiome affect tumor progression via the gut-to-brain axis (78).

To address these knowledge gaps, future research should focus on three key areas. First, longitudinal studies are needed to investigate how changes in gut microbiota correlate with the onset, severity, and treatment response of ITP over time (19). Second, mechanistic studies should explore the causal relationships between specific microbial taxa, microbial metabolites, immune dysregulation, and platelet homeostasis (47). Lastly, controlled clinical trials are essential to assess the therapeutic potential of microbiota-targeted interventions, including probiotics, prebiotics, dietary modifications, and FMT, in the management of ITP (11, 79).

By addressing these critical gaps, researchers can determine whether modulating the gut microbiota represents a viable therapeutic avenue for improving immune regulation and clinical outcomes in patients with ITP (80).

## 3 Gut microbiota modulation in ITP: a novel therapeutic strategy

Given the emerging evidence linking gut microbiota dysbiosis to immune dysregulation in ITP, researchers have begun exploring microbiota-targeted interventions as potential therapeutic strategies (19, 81). By restoring gut microbial balance, these approaches aim to modulate immune responses, promote immune tolerance, and mitigate the pathogenic mechanisms underlying ITP. Several microbiota-based interventions, including fecal FMT, probiotics, dietary modifications, and microbiome-targeted pharmacologic strategies, have shown promise in modulating the gut-immune axis (82, 83). However, the clinical translation of these therapies remains challenging due to a limited mechanistic understanding and the need for well-designed clinical trials.

Various microbiota-targeted therapies have been proposed as potential interventions for ITP, aiming to restore microbial balance and modulate immune function (11, 84). These strategies include FMT, probiotics, prebiotics, SCFA supplementation, and bile acid modulation (85, 86). While some have shown promise in autoimmune diseases, their application in ITP remains underexplored. The following **Table 2** summarizes the mechanisms, current evidence, and challenges associated with these microbiota-based therapies.

**Table 2:** Potential microbiota-targeted therapies for ITP.

Therapeutic Approach	Mechanism of Action	Current Evidence	Challenges & Limitations
Fecal Microbiota Transplantation (FMT)	Restores microbial diversity, promotes immune tolerance	Small studies suggest platelet improvement in ITP	Donor variability, safety concerns, regulatory issues
Probiotics	Enhances Treg activity, reduces inflammatory cytokines	Effective in other autoimmune diseases; underexplored in ITP	Strain specificity, inconsistent responses
Prebiotics	Supports beneficial bacterial growth, increases SCFA production	Some evidence in metabolic disorders and autoimmunity	Need for targeted prebiotics in ITP
SCFA Supplementation	Directly modulates immune responses via gut-immune axis	Butyrate shows promise in reducing inflammation	Bioavailability and dosing challenges
Bile Acid Modulation	Regulates T-cell differentiation and inflammatory pathways	Early-stage research in gut-immune interactions	Requires further validation in hematologic diseases

### 3.1 Fecal microbiota transplantation: mechanisms, clinical applications, and emerging evidence in ITP

FMT has gained attention as a promising approach to modulating gut microbiota composition (87). This procedure involves transferring fecal material from a healthy donor to a recipient, aiming to restore microbial diversity and improve immune homeostasis. Studies suggest that FMT can replenish beneficial taxa such as *Bacteroides* and *Firmicutes*, which are associated with immune tolerance (88). Originally developed for treating recurrent *Clostridioides difficile* infections, FMT has shown potential in various autoimmune and inflammatory diseases, including ITP (81).

**Figure 2:**
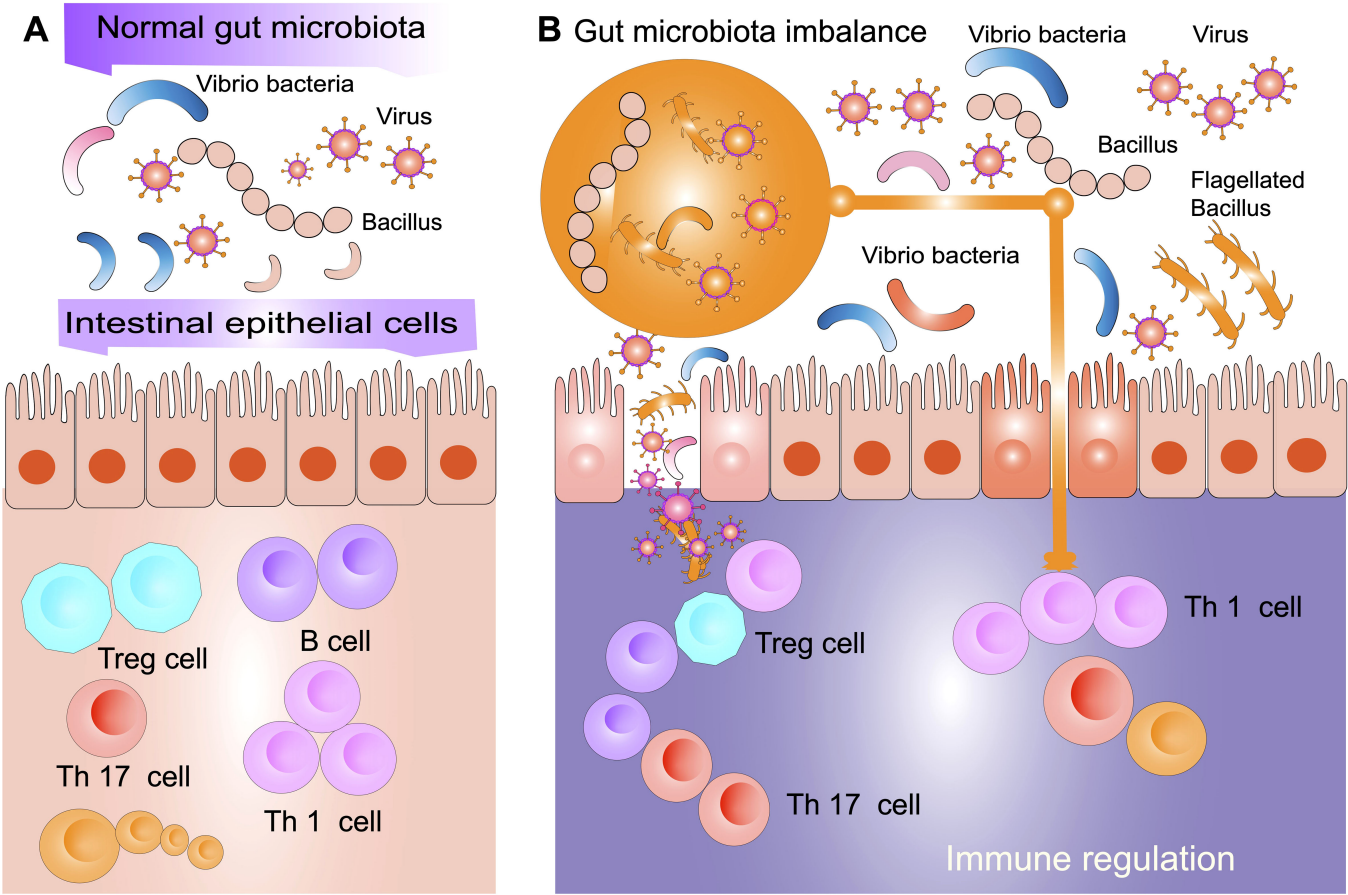
Gut microbiota balance and immune modulation. **(A)** In a normal gut microbiota environment, commensal bacteria such as *Vibrio* and *Bacillus* interact with intestinal epithelial cells to maintain immune homeostasis. This balance promotes the differentiation of regulatory T cells (Treg) and immune tolerance while preventing excessive inflammation driven by Th1 and Th17 cells. A balanced gut microbiota contributes to a well-regulated immune system by enhancing gut barrier integrity and modulating host immune responses. **(B)** In a state of gut microbiota imbalance (known as dysbiosis), there is an overrepresentation of pathogenic bacteria and viruses, along with a reduction in beneficial microbial populations. This microbial shift disrupts gut barrier function, leading to increased bacterial translocation and heightened immune activation. Dysbiosis skews immune regulation by reducing Treg activity and increasing Th1 and Th17 responses, promoting systemic inflammation and immune dysregulation. Such alterations in the gut microbiota may contribute to autoimmune conditions, including ITP, by exacerbating inflammatory pathways and impairing immune tolerance.

Restoring gut microbial diversity and modulating immune responses through fecal microbiota transplantation (FMT) has emerged as a promising therapeutic strategy to improve disease outcomes, as illustrated in **Figure 2**. This schematic highlights the key mechanisms through which FMT may contribute to immune homeostasis, including shifts in microbial composition, an increase in the activity of Tregs, and a reduction in systemic inflammation.

FMT modulates immune responses through several mechanisms, including the enhancement of Tregs, increased *Bifidobacterium* abundance, and suppression of pro-inflammatory Th1 and Th17 responses, as observed in autoimmune conditions such as multiple sclerosis and inflammatory bowel disease (IBD) (12, 46). Additionally, microbial metabolites such as SCFAs contribute to immune modulation by strengthening the intestinal barrier and suppressing excessive immune activation (89, 90).

The gut microbiota also influences systemic immune function, as demonstrated by recent findings on the gut-brain axis, where microbiota-derived metabolites, such as kynurenic acid, impact immune interactions at distant sites. This highlights the broader systemic effects of gut microbiota and underscores the need for further investigation into microbiota-targeted therapies in autoimmune conditions like ITP (78).

Emerging evidence suggests that gut microbiota may directly influence platelet regulation. Certain microbial metabolites, including SCFAs and secondary bile acids, have been shown to modulate platelet function by affecting megakaryocyte differentiation and platelet activation (11, 91). Moreover, gut dysbiosis has been associated with increased platelet aggregation and altered hemostatic balance in other diseases, suggesting that restoring microbial equilibrium through FMT could contribute to improved platelet homeostasis (92). While direct evidence of FMT’s effect on platelet regulation in ITP is limited, case reports and small clinical studies have observed platelet count improvement following microbiota restoration therapies (19, 72).

Despite these promising findings, several challenges remain in translating FMT into a standardized treatment for ITP. Donor microbiota variability and long-term engraftment pose significant challenges, as microbial compositions differ significantly between donors and recipients, potentially affecting therapeutic outcomes. Additionally, safety concerns include the risk of pathogen transmission, unintended immune activation, and unpredictable long-term effects (93). Therefore, well-controlled, randomized clinical trials are necessary to confirm the efficacy and safety of FMT in ITP management and to determine optimal protocols for donor selection, microbiota preparation, and recipient response monitoring. Addressing these issues is critical for integrating FMT into mainstream ITP treatment (88).

### 3.2 Probiotics and prebiotics: potential to restore gut balance and regulate immune responses

Probiotics and prebiotics represent another avenue for modulating gut microbiota in ITP (21, 47). Probiotics, including specific *Lactobacillus* and *Bifidobacterium* strains, enhance beneficial bacterial populations, while prebiotics, such as inulin and fructooligosaccharides, selectively stimulate microbial growth, promoting gut-immune homeostasis (94). However, the immunomodulatory effects of probiotics are highly strain-dependent, with some strains exerting potent anti-inflammatory properties, while others may have limited or even opposing effects in different individuals (95).

Clinical studies suggest that *Lactobacillus rhamnosus* and *Bifidobacterium breve* increase Treg activity while reducing IL-6 and TNF-α, leading to decreased systemic inflammation in autoimmune diseases (96). Additionally, *Lactobacillus plantarum* has been shown to enhance gut barrier integrity by upregulating tight junction proteins, thereby preventing bacterial translocation and reducing systemic immune activation (97). Similarly, prebiotics, including inulin and fructooligosaccharides, serve as metabolic substrates for beneficial bacteria, fostering a gut environment that supports immune homeostasis (98). The combination of specific probiotic strains with targeted prebiotic supplementation (synbiotics) may offer enhanced therapeutic potential by optimizing microbial colonization and metabolic activity (99).

Despite promising preclinical and clinical evidence supporting probiotic use in other autoimmune diseases, their application in ITP remains underexplored (100). One of the major challenges is the variability in probiotic efficacy, which stems from strain-specific effects, inter-individual differences in microbiota composition, and inconsistencies in host immune responses (101). For instance, while *Bifidobacterium adolescentis* has been shown to reduce Th17-mediated inflammation in rheumatoid arthritis, similar effects have not been confirmed in ITP (102). Additionally, the interaction between probiotics and endogenous microbial communities can lead to variable colonization success, limiting the predictability of therapeutic outcomes (103).

Future research should focus on defining optimal probiotic formulations, identifying microbial signatures predictive of response, and evaluating their impact on platelet homeostasis and immune regulation (69). Advancements in microbiome sequencing and metabolomic profiling may enable the development of personalized microbiota-based interventions, tailoring probiotic and prebiotic therapies to individual ITP patients for improved efficacy (104).

### 3.3 Dietary and metabolomic interventions: role of SCFAs, bile acids, and tryptophan metabolites in immune modulation

Dietary interventions play a pivotal role in shaping gut microbiota composition and function (87). Specific dietary components influence the production of key microbial metabolites, which, in turn, modulate immune responses (105). Among these, SCFAs, bile acids, and tryptophan-derived metabolites are particularly significant in maintaining immune homeostasis and resolving inflammation (105). However, while their immunoregulatory effects have been well characterized in autoimmune diseases, their specific impact on platelet regulation in ITP remains an emerging area of research.

SCFAs, including butyrate and propionate, are microbial fermentation products that exert profound immunomodulatory effects (53). These metabolites enhance regulatory T cell (Treg) differentiation and inhibit pro-inflammatory cytokines such as IL-6, TNF-α, and IFN-γ, thereby reducing autoimmune activity (106). Butyrate, in particular, acts as a histone deacetylase inhibitor, promoting Treg expansion and suppressing Th17-mediated inflammation, mechanism relevant to platelet destruction in ITP (69, 107). Additionally, SCFAs strengthen gut barrier integrity by upregulating tight junction proteins, thereby reducing microbial translocation and systemic immune activation factors that may contribute to excessive immune responses in ITP (62, 69).

Recent studies suggest that lower SCFA levels in autoimmune conditions are linked to immune dysregulation and altered thrombopoiesis (108, 109). Butyrate has been shown to reduce megakaryocyte apoptosis, potentially impacting platelet production and turnover (110). Additionally, SCFA supplementation has demonstrated protective effects on platelet homeostasis in inflammatory conditions, indicating a possible therapeutic avenue for ITP (32, 108).

Bile acids, traditionally recognized for their role in lipid metabolism, have recently emerged as key modulators of immune responses, influencing T-cell differentiation via interactions with gut microbiota (111). Secondary bile acids, such as deoxycholic acid and lithocholic acid, regulate immune pathways through host receptors, including the Farnesoid X receptor (FXR) and Takeda G-protein-coupled receptor 5 (TGR5) (112). FXR activation suppresses Th17 cell differentiation, thereby reducing inflammatory cytokine production, a process that could mitigate platelet autoantibody formation in ITP (113). TGR5 signaling has been shown to enhance Treg function, contributing to immune tolerance and reducing autoimmunity in conditions such as SLE and RA (8, 114).

Alterations in bile acid metabolism have been identified in ITP patients, suggesting a potential role in immune dysregulation (47). Recent studies indicate that bile acid supplementation can modulate thrombopoiesis, potentially linking gut microbiota-derived bile acids to platelet production and function (115). These findings support the investigation of bile acid-targeted therapies in ITP as a novel immunomodulatory approach (11).

Tryptophan metabolism also plays a crucial role in immune homeostasis, with its metabolites modulating immune responses via the aryl hydrocarbon receptor (AhR) and indoleamine 2,3-dioxygenase (IDO) pathways (116). Kynurenine, an AhR ligand, has been shown to promote Treg differentiation, enhancing immune tolerance and reducing autoimmune activity (117). Indole derivatives regulate Th17 differentiation, thereby controlling inflammatory responses that may drive platelet destruction in ITP (2, 118). Additionally, serotonin, a tryptophan metabolite, has been implicated in platelet aggregation, further underscoring the potential gut-immune-thrombosis axis in ITP (11, 43). Dysregulation of tryptophan metabolism in autoimmune conditions suggests a possible link to ITP pathophysiology, with recent findings highlighting microbiota-mediated tryptophan metabolism as a key factor in hematologic disorders (119).

Despite growing interest in dietary interventions, their role in ITP remains speculative (120). Limited clinical data exist regarding the effects of specific dietary modifications on platelet counts and immune function in ITP patients (121). Additionally, dietary interventions may have variable effects depending on individual microbiota composition and metabolic responses (122). Future research should prioritize characterizing microbial metabolite profiles in ITP patients to identify potential therapeutic targets, investigating the roles of SCFAs, bile acids, and tryptophan metabolites in modulating platelet regulation and immune responses. It should also assess the clinical efficacy of dietary interventions, including probiotic and prebiotic supplementation, as well as bile acid modulation, through well-designed randomized controlled trials (47, 123). By integrating microbiome sequencing and metabolomic profiling, researchers can better define the role of microbial metabolites in platelet function and immune modulation, leading to potential novel therapeutic approaches in ITP (52, 79).

### 3.4 Pharmacologic strategies targeting the gut: microbiome-based drug development

Pharmacologic approaches to modulating the gut microbiota are emerging as a potential strategy for treating autoimmune diseases, including ITP (12). These approaches include microbiome-based small molecules, postbiotics, and engineered probiotics designed to selectively modulate microbial communities and immune function (21).

Postbiotics, which are bioactive compounds produced by beneficial bacteria, have shown promise in immune modulation without the need for live microorganisms (124). Microbiome-based small molecules are being developed to target specific microbial metabolic pathways that influence immune responses (125). Additionally, engineered probiotics are being designed to deliver immunomodulatory molecules directly within the gut, offering a targeted approach to restoring immune balance (126).

While microbiome-based drug development is still in its early stages, these strategies hold great potential for providing precise and effective treatments for ITP (127). However, key challenges include ensuring microbial stability, understanding long-term safety, and optimizing drug delivery systems (128). Future studies should investigate the pharmacokinetics and pharmacodynamics of these microbiome-based therapies in ITP, as well as their potential for integration with standard treatments (129).

## 4 Challenges and limitations in gut microbiota therapy for ITP

Despite the growing interest in microbiota-targeted therapies for ITP, several challenges and limitations must be addressed before these strategies can be effectively translated into clinical practice. While preclinical and early clinical studies suggest that microbiota modulation may help restore immune tolerance and regulate platelet homeostasis (130), inconsistencies in research findings, methodological limitations, and unresolved safety concerns remain key barriers. This section critically examines the current knowledge, debates ongoing controversies, and identifies gaps that future research should address.

### 4.1 Variability in gut microbiota composition among individuals

One of the fundamental challenges in microbiota-based therapies is the high degree of inter-individual variability in gut microbial composition (131). Factors such as genetics, diet, medication history (including prior antibiotic use), and environmental influences contribute to significant differences in microbial diversity and function (132). This variability complicates the standardization of microbiota-based interventions, as a treatment effective for one patient may not yield similar benefits for another (133). Additionally, baseline microbiota differences may influence therapeutic responses, highlighting the need for patient-specific approaches. Future research should focus on stratifying patient populations based on microbiome profiling to optimize treatment efficacy (134).

### 4.2 Standardization and safety concerns in FMT and microbiota-based interventions

FMT has shown promise as a microbiota-based intervention in autoimmune diseases, but its application in ITP remains largely experimental (135). One major concern is the lack of standardization in FMT protocols, including donor selection, preparation methods, and delivery routes (87). Donor microbiota composition can vary significantly, leading to inconsistent therapeutic outcomes (136). Additionally, potential risks associated with FMT include the transmission of infectious agents, unintended immune activation, and long-term alterations in gut microbiota that may have unpredictable consequences (137).

Beyond FMT, the safety profile of probiotics and prebiotics in ITP patients has not been rigorously evaluated (69). While some probiotic strains exhibit immunomodulatory properties, others may provoke excessive immune activation or lead to bacterial overgrowth, particularly in immunocompromised individuals (138). To address these safety concerns, further clinical trials with well-defined protocols are needed to assess the risks and benefits of microbiota-targeted therapies in ITP.

### 4.3 Need for robust clinical trials and biomarker discovery in ITP-microbiota research

Although emerging studies suggest a link between gut dysbiosis and ITP, most existing research relies on small-scale, cross-sectional studies with limited statistical power (139). Longitudinal studies are needed to investigate how changes in gut microbiota correlate with the onset, severity, and treatment response of ITP over time. Second, mechanistic studies should explore the causal relationships determine whether gut microbiota alterations precede ITP onset or arise as a consequence of the disease and its treatments (18). Furthermore, the identification of reliable microbial biomarkers for disease progression and treatment response remains a significant gap in current research (140). Developing standardized methods for microbiome analysis, including metagenomic sequencing and metabolomic profiling, could help establish microbiota-based diagnostic and prognostic tools for ITP (141).

Clinical trials evaluating microbiota-targeted interventions in ITP are also lacking (52). While probiotics, prebiotics, dietary modifications, and FMT have been explored in other autoimmune conditions, few studies have directly assessed their impact on platelet counts and immune regulation in ITP patients. Large-scale, randomized controlled trials are essential to determine the efficacy, safety, and durability of these interventions in a hematologic context (142).

### 4.4 Ethical and regulatory considerations in applying microbiota therapies to hematologic disorders

The integration of microbiota-based therapies into hematologic disease management raises several ethical and regulatory challenges (143). Unlike conventional pharmacologic agents, microbiota-based interventions involve live organisms, making it difficult to define consistent dosing, manufacturing processes, and quality control standards (69). Regulatory agencies, such as the FDA and EMA, currently classify FMT as an investigational therapy, necessitating rigorous oversight before its widespread adoption in ITP treatment (77).

Additionally, ethical concerns related to FMT donor selection, consent processes, and long-term safety monitoring must be addressed (87). Patients undergoing microbiota-based treatments should be informed of potential risks, including unforeseen immune complications or persistent microbiome alterations (144). Establishing regulatory frameworks that balance innovation with patient safety will be crucial in advancing microbiota-targeted therapies in ITP (129).

To overcome these limitations, future research should focus on developing standardized microbiota-based protocols by establishing guidelines for donor screening, sample preparation, and treatment administration to improve the reproducibility and safety of microbiota-based therapies (93). Additionally, personalizing microbiota interventions through microbiome sequencing and precision medicine approaches can help tailor treatments based on individual microbiota profiles, thereby increasing therapeutic efficacy (145). Conducting large-scale, controlled clinical trials will be essential for evaluating the long-term impact of microbiota-targeted therapies in ITP and ensuring their clinical adoption (84). Furthermore, clarifying the mechanisms of action through further research is necessary to elucidate how specific microbial taxa and metabolites influence platelet regulation and immune responses in ITP (11). Addressing these aspects will provide a stronger foundation for the integration of microbiota-based therapies into ITP management (96). By tackling these challenges and knowledge gaps, microbiota-targeted interventions may become a viable and evidence-based approach for managing ITP, complementing existing immunomodulatory treatments and improving patient outcomes.

## 5 Future directions and clinical translation

While significant strides have been made in understanding the gut-immune axis in ITP, translating these findings into effective clinical applications remains a challenge (18). Several critical gaps must be addressed, including the lack of standardized microbiota profiling in clinical practice, variability in treatment responses, and the need for robust clinical trials to validate microbiota-based interventions (146). This section highlights the key areas of future research that could bridge these gaps and facilitate the integration of microbiota-targeted therapies into mainstream ITP management.

### 5.1 Integrating microbiota profiling in ITP diagnosis and prognosis

One promising avenue for advancing ITP management is the incorporation of microbiota profiling into diagnostic and prognostic assessments. Given the increasing evidence that gut microbiota composition influences immune responses and disease severity, recent studies have identified microbial signatures linked to hematologic diseases, where altered *Bacteroides* and *Enterobacteriaceae* profiles predict disease progression, suggesting potential biomarker applications for ITP (19, 147).

Recent advancements in microbiome sequencing have facilitated the identification of microbial alterations linked to systemic disease progression (140). For instance, studies in brain metastases have demonstrated how gut microbiota composition can influence disease dynamics, underscoring the potential of microbiota profiling as a valuable tool for diagnosing and monitoring immune-mediated disorders such as ITP (78). Metagenomic sequencing enables high-resolution characterization of microbial taxa, revealing microbiota-related risk factors predictive of autoimmune disease severity (148).

**Figure 3:**
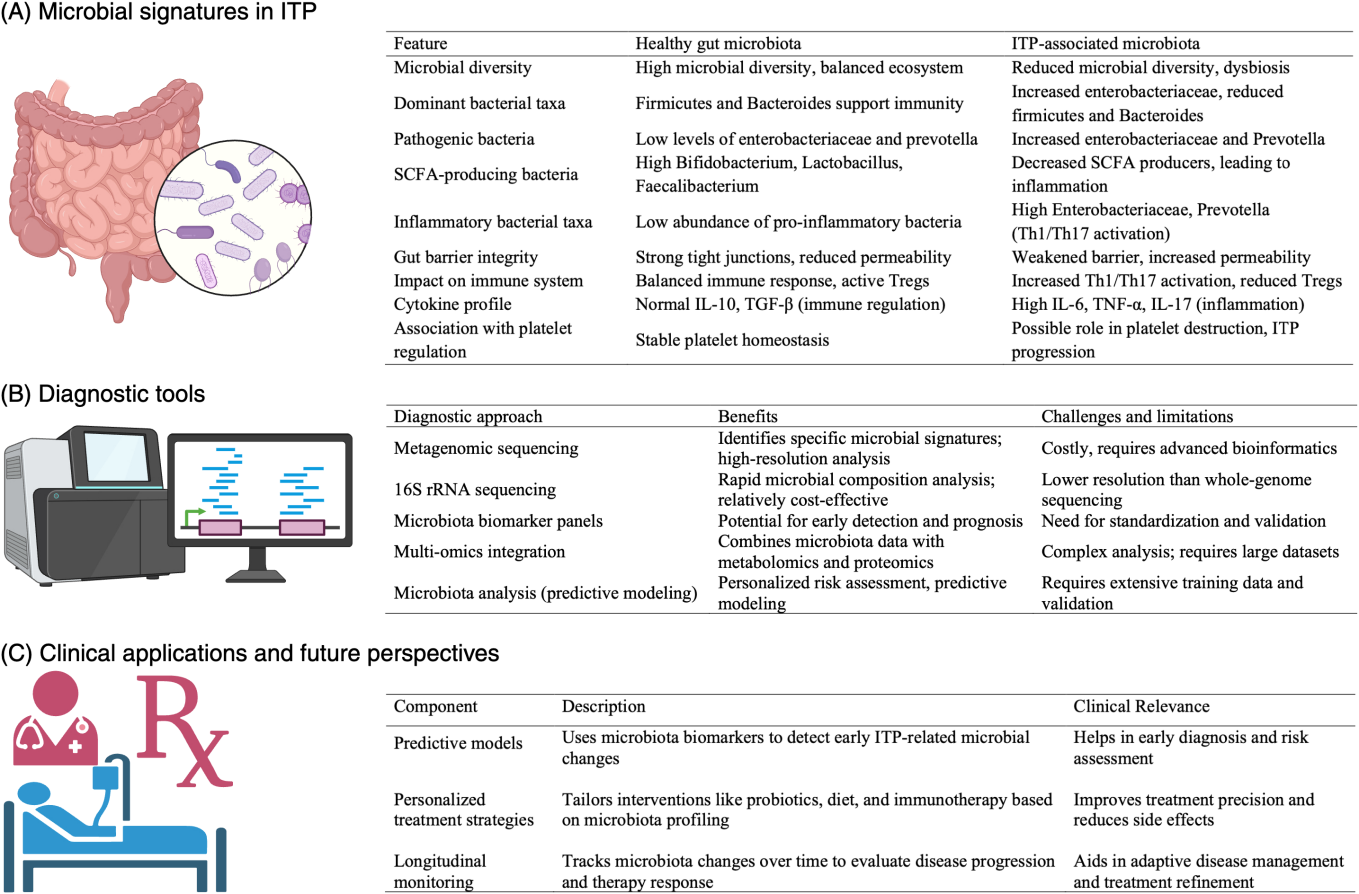
Overview of microbiota-based biomarkers and diagnostic approaches in ITP. This figure illustrates the role of microbiota profiling in the diagnosis and management of immune thrombocytopenia (ITP). **(A)** Microbial signatures in ITP highlight key alterations in bacterial taxa, including decreased *Bacteroides* and *Firmicutes*, and increased *Enterobacteriaceae*, which are associated with immune dysregulation and platelet destruction. **(B)** A comparison of diagnostic tools—metagenomic sequencing, 16S rRNA sequencing, microbiota biomarker panels, and multi-omics integration—highlighting their benefits and limitations for clinical application in ITP. **(C)** Clinical applications and future perspectives include predictive models for early detection, personalized treatment strategies integrating probiotics and dietary interventions, and longitudinal monitoring to assess disease progression and therapeutic responses. This schematic underscores the potential of microbiota-based diagnostics in improving precision medicine approaches for ITP management.

An overview of microbiota-based biomarkers and diagnostic approaches in ITP, including key microbial alterations, sequencing methodologies, and their potential clinical applications, is presented in **Figure 3**. This schematic highlights how microbiota profiling could be integrated into routine diagnostic workflows to enhance precision medicine approaches in ITP.

Despite these advances, specific microbial signatures distinguishing ITP from other autoimmune and hematologic diseases remain unclear. While some studies suggest that gut dysbiosis may contribute to platelet regulation and immune modulation in ITP, no definitive microbial biomarkers have been validated for ITP diagnosis or disease stratification (11, 19). Furthermore, the high inter-individual variability in gut microbiota composition complicates the reproducibility and clinical application of microbiome-based diagnostics (12, 131).

The application of microbiota profiling in ITP diagnosis presents both opportunities and challenges (18, 19). **Table 3** summarizes the advantages and limitations of various microbiome-based diagnostic approaches, highlighting their potential role in disease monitoring and personalized treatment strategies.

**Table 3:** Advantages and challenges of microbiota profiling in ITP diagnosis and prognosis.

Diagnostic Tool	Benefits	Challenges & limitations
Metagenomic Sequencing	Identifies specific microbial signatures, high-resolution analysis	Costly, requires specialized bioinformatics
16S rRNA Sequencing	Rapid microbial composition analysis, relatively cost-effective	Lower resolution than whole-genome sequencing
Microbiota Biomarker Panels	Potential for early detection and prognosis	Need for standardization and validation
Microbiota Analysis	Personalized risk assessment, predictive modeling	Requires extensive training data and validation

While microbiota profiling holds promise, its clinical relevance in ITP remains uncertain. Studies suggest an association between gut dysbiosis and platelet regulation in ITP, but causal mechanisms remain unclear, necessitating longitudinal studies to establish microbiota-driven disease modulation (19, 47). Additionally, the standardization of sampling methods, data interpretation, and validation across different populations is needed before microbiota profiling can be reliably incorporated into clinical practice (149).

To advance microbiota profiling as a diagnostic tool in ITP, future research should focus on identifying robust microbial biomarkers specific to ITP that distinguish it from other hematologic and autoimmune disorders (150); conducting large-scale, multi-cohort microbiome studies to evaluate consistent microbial signatures across diverse populations (151); integrating microbiome data with clinical parameters and multi-omics approaches (e.g., metabolomics, proteomics) to refine diagnostic accuracy (152); and developing standardized guidelines for microbiome-based clinical diagnostics to ensure reproducibility and regulatory approval for ITP diagnosis (153).

### 5.2 Personalized microbiota-based therapies for ITP management

The concept of personalized microbiota-based therapies is gaining traction as an alternative or adjunctive strategy for ITP management (19). Given the variability in microbiota composition among individuals, tailoring interventions based on a patient’s specific microbial profile may enhance treatment efficacy (82). Personalized approaches may include selective probiotic formulations, prebiotic-enriched diets, or customized FMT protocols designed to restore microbial balance and immune homeostasis (154).

However, significant challenges remain. The efficacy of probiotics and prebiotics in ITP has not been rigorously tested, and the optimal strains or formulations for modulating immune responses remain undefined (138). Moreover, inter-individual differences in microbiota composition may influence therapeutic outcomes, necessitating a precision medicine approach (145). Future studies should focus on characterizing microbiota profiles associated with positive treatment responses and developing predictive models to guide personalized microbiome-based interventions (155).

### 5.3 Advances in microbiome sequencing and artificial intelligence for targeted interventions

Technological advancements in microbiome sequencing and artificial intelligence (AI) are revolutionizing the development of microbiota-targeted interventions. High-throughput sequencing techniques, such as 16S rRNA sequencing and shotgun metagenomics, allow for comprehensive profiling of gut microbial communities, facilitating the identification of microbial alterations linked to ITP (156). These tools could be leveraged to refine microbiota-based diagnostics and therapeutic strategies.

AI-driven approaches further enhance our ability to analyze complex microbiota datasets, predict treatment responses, and develop targeted therapeutic interventions (157). Machine learning models can identify microbial patterns associated with disease states and recommend tailored microbiota-based therapies based on an individual’s gut microbiota composition. Integrating AI with microbiome research holds great potential for optimizing precision medicine approaches in ITP and improving clinical outcomes (158).

### 5.4 Potential for gut microbiota manipulation in combination with standard ITP therapies

Combining microbiota-targeted therapies with conventional ITP treatments represents an exciting avenue for improving patient outcomes (159). Current standard therapies, such as corticosteroids, thrombopoietin receptor agonists, and immunosuppressive agents, exhibit variable efficacy and often associated with significant side effects (160). Modulating the gut microbiota may serve as a complementary strategy to enhance treatment efficacy, reduce immune-related side effects, and improve long-term disease management (69).

Certain microbiota-targeted interventions, such as probiotics and SCFA-based dietary strategies, may help regulate immune responses, reduce inflammation, and promote platelet production, thereby decreasing reliance on long-term immunosuppressive therapies (108). Additionally, microbiota-based strategies may facilitate immune tolerance in refractory ITP cases, improving the likelihood of sustained remission. However, the challenge remains in identifying the most effective microbiota-based combinations and understanding their interactions with existing therapies. Future clinical trials should investigate the synergistic effects of microbiota-targeted therapies with current ITP treatments to optimize patient care (129).

Future directions should prioritize robust Phase II/III clinical trials, standardized microbiome diagnostics, longitudinal microbiota-immune tracking, and integration of AI-driven predictive models to personalize treatment. These efforts will advance precision microbiome therapeutics for ITP and improve long-term outcomes.

While microbiota-targeted therapies hold great promise for ITP, several obstacles must be addressed before they can be effectively implemented in clinical practice (161). Future research should prioritize large-scale, randomized controlled trials to validate the efficacy of microbiota-based interventions, establish standardized diagnostic protocols, and further explore the mechanistic pathways linking gut dysbiosis to ITP pathogenesis (162). Integrating microbiota profiling into precision medicine approaches and leveraging AI-driven strategies may pave the way for innovative and personalized treatment options (145).

Robust Phase II/III clinical trials are urgently needed to validate the clinical efficacy of microbiota-targeted interventions, including fecal microbiota transplantation, SCFA supplementation, and strain-specific probiotics (163). These studies should incorporate standardized microbiome profiling and immune monitoring to establish reproducible outcomes (164). In parallel, recent proposals emphasize the importance of prospective longitudinal studies and gnotobiotic mouse models to establish causality and elucidate the underlying microbial mechanisms that regulate immune responses and platelet homeostasis in ITP (165).

By addressing these challenges, the field can move toward more targeted and effective microbiota-based therapies for ITP, ultimately improving patient outcomes and transforming disease management (127).

## 6 Conclusion

The growing recognition of the gut-immune axis in primary ITP represents a paradigm shift in understanding and managing this autoimmune disorder (19). Traditional treatment strategies have primarily focused on immunosuppressive approaches; however, emerging evidence highlights the pivotal role of gut microbiota in immune regulation and disease progression (12). The interplay between gut microbiota, immune tolerance, and platelet homeostasis offers new avenues for therapeutic intervention, shifting the focus toward microbiota-targeted strategies.

Microbiota-based therapies, including FMT, probiotics, prebiotics, and dietary interventions, hold significant promise in modulating immune responses and restoring microbial balance in ITP (166). These approaches can enhance immune tolerance, reduce inflammation, and complement existing treatment modalities (167). However, while early studies provide compelling insights, further clinical trials are essential to validate the safety, efficacy, and long-term effects of these interventions (168).

Despite these promising developments, several challenges must be addressed before microbiota-targeted therapies can be fully integrated into clinical practice. Standardizing microbiome profiling methods, identifying reliable microbial biomarkers, and optimizing therapeutic strategies tailored to individual microbiota compositions remain critical research priorities (154, 169). Importantly, clarifying the temporal relationship between dysbiosis and immune dysfunction in ITP requires longitudinal microbiome studies and mechanistic experiments, including the use of gnotobiotic animal models (165). Additionally, long-term studies are needed to evaluate the durability of microbiota modulation and its sustained effects on disease outcomes.

Continued research is crucial for translating gut-immune insights into effective clinical strategies. Advancements in microbiome sequencing, artificial intelligence-driven microbiota profiling, and biomarker discovery may facilitate the development of personalized, precision-based therapies for ITP (104). Integrating microbiota modulation with standard immunosuppressive therapies and thrombopoietin receptor agonist therapies may lead to synergistic treatment effects and improved patient outcomes (82).

In conclusion, leveraging the gut-immune axis for ITP management represents an exciting frontier in autoimmune disease research. As our understanding of gut microbiota expands, microbiota-targeted interventions may pave the way for more effective, sustainable, and personalized treatment strategies for ITP, ultimately improving patients’ quality of life and long-term disease management.
